# An atypical bilateral trifurcation of recurrent laryngeal nerve

**DOI:** 10.1186/s12893-022-01624-w

**Published:** 2022-05-13

**Authors:** P. B. Krishnan, M. P. Santosh

**Affiliations:** Rajarajeswari Medical College and Hospital, Kambipura Mysore Road, Bangalore, India 560074

**Keywords:** Thyroidectomy, Recurrent laryngeal nerve variations, Multinodular goitre, RLN trifurcations

## Abstract

**Background:**

Thyroidectomy is a frequently performed surgery for benign and malignant conditions. Nevertheless, one of the most critical complications of thyroidectomy is recurrent laryngeal nerve (RLN) injury leading to vocal cord paralysis. A thorough knowledge of the anatomical variations of RLN and ligation of the related vessels close to their distal branches is critical to avoid injury.

**Case presentation:**

Here, we report the first case of bilateral trifurcation of recurrent laryngeal nerve (RLN) in a 40-year old woman with multinodular goitre. Total thyroidectomy was performed and RLN was preserved bilaterally. Followed by a precise dissection, fine branches were traced penetrating the larynx. We did not observe any further post-operative complications and patient was discharged with desired outcomes.

**Conclusions:**

Anatomical variations of the RLN include—bifurcations, trifurcations, relation of RLN with inferior thyroid artery (ITA) and presence of non-recurrent laryngeal nerve. Only RLN dividing at a distance greater than 5 mm (branching point distance) before its entry into the larynx beneath the cricothyroid are said to bifurcate or trifurcate. Approximately 25% of nerves show branching [71%—unilateral and 18%—bilateral bifurcation]. Incidence of unilateral trifurcations have been noted be 0.9% and the rates of bilateral trifurcation and the divisions of the branches is yet to be ascertained. This is the first report of a bilateral trifurcation of RLN, detected in patient with multinodular goitre and hence warrants a precise analysis of variations of the RLN in patients undergoing thyroidectomy, which is critical to prevent RLN injury.

## Background

The Recurrent Laryngeal Nerve (RLN) arises as a branch from the vagus nerve and terminates at the larynx carrying both sensory and motor fibres. The RLN serves as the main motor nerve for all intrinsic laryngeal muscles, with the exception of the cricothyroid. The RLN loops deep into the relevant vessels, follows the tracheoesophageal groove and enters the larynx into the inferior pharyngeal constrictor muscle. The RLN injury is a major concern during total thyroidectomy. Unilateral RLN injury leads to partial vocal cord paralysis and hoarseness, while bilateral injury results in stridor or acute airway obstruction [1]. Approximately 3–8% of cases suffer from transient post-operative paralysis and 0.3- 3% of cases end up with permanent paralysis [1]. In a study by Beneragama et al. a total of 213 (right = 114, left = 99) RLN in 137 patients were examined. Seventy-seven (36%) nerves bifurcated or trifurcated before entry into the larynx. Bifurcations were more common on the right (43%) than on the left (28%) (P = 0.05). Trifurcations were seen in eight nerves, seven on the right and one on the left (P = 0.05) [2]. However bilateral trifurcations were not observed. Understanding anatomical variations of nerve—bifurcations, trifurcations, relation to inferior thyroid artery and presence of non-RLN helps in reducing the incidence of injury.

Precise knowledge of anatomical variations of the recurrent laryngeal nerve (RLN) is essential to prevent inadvertent intraoperative injury. Here we present, a unique bilateral trichotomy of the RLN anatomical variability pertaining to its course, branching pattern, and relationship to the inferior thyroid artery.

## Case presentation

A 40-year old woman with a medical history of 6-months of difficulty in swallowing presented with a thyroid swelling in the neck. Patient did not have any features of hyper/hypo-thyroidism and no co-morbidities. Vitals were within normal limits. Local examination revealed a butterfly shaped swelling in the neck, which moved with deglutition. Clear palpable nodules were apparent in either lobes of the thyroid gland.

Thyroid hormones and free thyroxine levels were within normal limits. Ultrasonography of neck showed bulky thyroid lobes with multiple nodules features suggestive of multinodular goitre. Fine needle aspiration cytology was also suggestive of multi nodular goitre. Total thyroidectomy was performed.

**Fig. 1:**
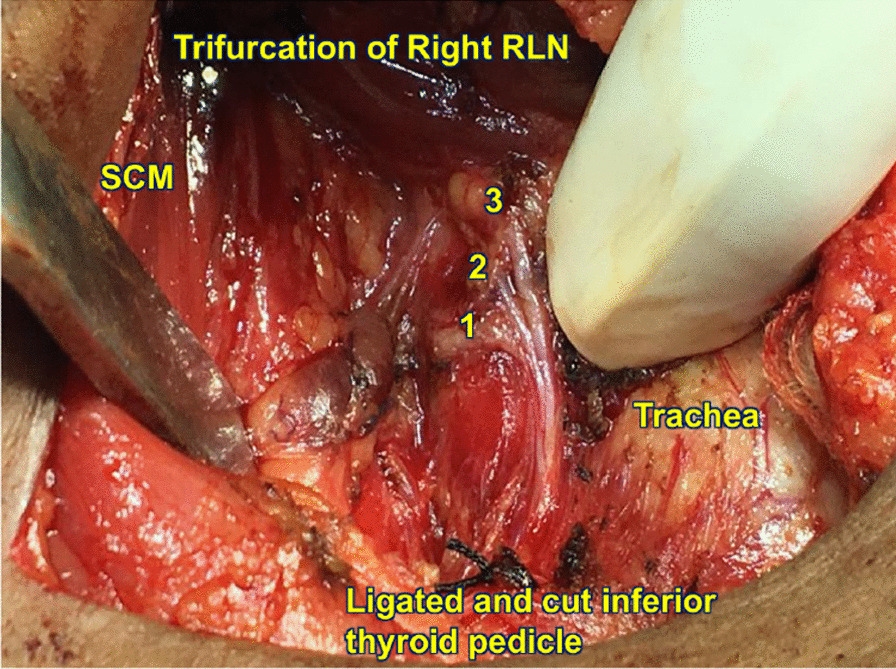
Trifurcation of right recurrent laryngeal nerve in the tracheoesophageal groove as it passes through the larynx. Numbers refer to the three branches of RLN

**Fig. 2:**
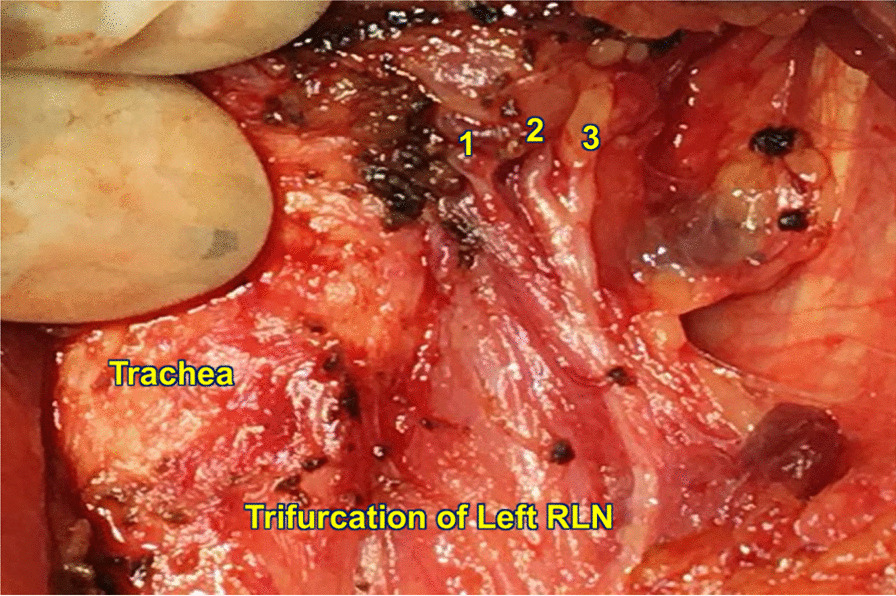
Trifurcation of left recurrent laryngeal nerve in the tracheoesophageal groove as it enters the larynx. Numbers refer to the three branches of RLN

In exploration RLNs were identified in the tracheoesophageal groove. Precise dissections performed on either side revealed, bilaterally the RLN divided into three branches (Fig. 1, 2) at a distance of 10 mm from cricoid cartilage. Clinical analysis revealed multinodular goitre and post-operative period was uneventful.

## Discussion and conclusion

Thyroidectomy is a common surgery performed for various conditions of the thyroid gland. Potential complications of thyroid surgery include bleeding, injury to the RLN, hypoparathyroidism, hypothyroidism, and infection. The RLN injury is common concern during total thyroidectomy and is considered a challenge even for the most experienced surgeons. This is due to multiple anatomical variations of RLN encountered intra operatively.

On the right side, the RLN originates from the vagus nerve as it crosses anterior to the subclavian artery. The RLN passes inferior and then posterior to the right subclavian artery and ascends in a position lateral to the trachea along the tracheoesophageal groove. During thyroidectomy, at the level of the lower border of the thyroid, the right RLN can usually be found within 1 cm lateral to or within the tracheoesophageal groove [3]. Compared with the left side, the course of the RLN on the right is less predictable in the lower portions of the field of thyroid surgery and follows a more oblique course. However, as it ascends to the midportion of the thyroid, the right RLN assumes its position within the tracheoesophageal groove. Usually the nerve is found immediately anterior or posterior to a main arterial trunk of the inferior thyroid artery at this level [3].

On the left side, the RLN separates from the vagus as that nerve passes anterior to the arch of aorta. The left RLN passes inferior and posteromedial to the aorta at the ligamentum arteriosum and begins to ascend toward the larynx. The left RLN enters the tracheoesophageal groove as it ascends to the level of the lower pole of the thyroid [3]. Compared with the right RLN, the left RLN is more predictably directly within the tracheoesophageal groove in the lower portions of the surgical field for thyroidectomy. Both RLNs are consistently found within the tracheoesophageal groove when they are within 2.5 cm of their entrance into the larynx. These nerves pass either anterior or posterior to a branch of the inferior thyroid artery (ITA) and enter the larynx at the level of the cricothyroid articulation on the caudal border of the cricothyroid muscle [3].

Visible anatomic variations include extra-laryngeal branching such as bifurcations, trifurcations, relation of RLN with inferior thyroid artery (ITA) & presence of non-recurrent laryngeal nerve [4]. The incidence of non-recurrent RLN is about 0.5–1.5% most commonly associated with arterial anomalies most commonly an aberrant right subclavian artery, also known as arteria lusoria [3]. The relationship between the RLN and the ITA is crucial and serves as a landmark for identification of the RLN and its branches during surgery. The RLN is visible at the level of ITA & hence nerve dissection should be started distally and branches should be traced as they enter the larynx. A study by Makay et al. identified sixteen variations of the nerve deep to the artery [5].

The RLN dividing at a distance greater than 5 mm (branching point distance) before its entry into the larynx beneath the cricothyroid are said to bifurcate or trifurcate [2]. Approximately 25% of nerves show branching [71%—unilateral and 18%—bilateral bifurcation [3, 6]. In a bifurcation anterior branch is motor to all intrinsic muscles of the larynx except the cricothyroid, and posterior cricoarytenoid (PCA). The posterior branch provides motor fibres to the PCA and is sensory to laryngeal mucosa [7]. Incidence of unilateral trifurcations have been noted be 0.9% in a series involving 2626 nerve dissections [8], however the rates of bilateral trifurcation and the divisions of the branches is yet to be ascertained.

The overall incidence of permanent and transient paralysis of RLN have been reported to be 0.3–3% and 3–8% [1]. Clamping, thermal effect, inappropriate dissection & ligation are considered as the main reasons for paralysis of RLN. Intraoperative nerve monitoring using electromyography signals helps to identify the nerve and preserve the branches thereby reducing the incidence of RLN injury [9]. Our report on a unique case of bilateral trifurcation adds to the existing findings as a novel variant of the RLN.

This study may have implications for surgical techniques and consideration of an appropriate approach for preserving the extra-laryngeal branches of the RLN during thyroidectomy. Therefore, a comprehensive knowledge of anatomical variations in the branching pattern of the RLNs, are indispensable to prevent RLN injury.

## Data Availability

Data sharing is not applicable to this article, as no datasets were generated or analysed during the current study.

## Abbreviations

PCA: Posterior cricoarytenoid
RLN: Recurrent laryngeal nerve
ITA: Inferior thyroid artery
